# Efficacy and safety of once-weekly semaglutide monotherapy in a young subject with Prader-Willi syndrome, obesity, and type 2 diabetes: a case report

**DOI:** 10.3389/fendo.2025.1533209

**Published:** 2025-02-10

**Authors:** Elisa Dinoi, Giuseppe Daniele, Angela Michelucci, Fulvia Baldinotti, Fabrizio Campi, Piero Marchetti, Stefano Del Prato, Angela Dardano

**Affiliations:** ^1^ Department of Clinical and Experimental Medicine, University of Pisa, Pisa, Italy; ^2^ Center for Instrument Sharing of the University of Pisa (CISUP), Pisa, Italy; ^3^ Laboratory of Molecular Genetics, University Hospital of Pisa, Pisa, Italy; ^4^ Section of Diabetes and Metabolic Diseases, Azienda Ospedaliero-Universitaria Pisana, Pisa, Italy; ^5^ Interdisciplinary Research Center “Health Science”, Sant’Anna School of Advanced Studies, Pisa, Italy

**Keywords:** Prader-Willi syndrome, obesity, type 2 diabetes mellitus, GLP-1 RA, semaglutide, case report

## Abstract

**Background:**

The treatment of obesity and type 2 diabetes (T2D) in Prader-Willi syndrome (PWS) is still a challenge. Glucagon-like peptide 1 receptor agonists (GLP-1 RA) are attractive options, since they effectively reduce weight and improve blood glucose, without increasing the risk of hypoglycemia. However, data on their use in PWS are scarce.

**Case description:**

In 2019, a 27-year-old male came to our Clinic because of first appearance of severe hyperglycemia (fasting plasma glucose 22.5 mmol/L). Based on clinical presentation, PWS was suspected, and diagnosis was confirmed by genetic tests. The patient was discharged on a basal-bolus insulin therapy managed by his parents due to his cognitive impairment. In spite of COVID-19 pandemic, the patient achieved tight glycemic control (HbA1c 41 mmol/mol) with non-severe hypoglycemic events in the face of significant body weight (BW) increase (+ 13 kg vs baseline). Insulin therapy was then discontinued, and once-weekly semaglutide (up to 0,5 mg weekly) was started. At 12-month follow-up, BW dropped from 79 to 73 kg while maintaining excellent glycemic control (HbA1c 40 mmol/mol). At 24-month follow-up, glycemic control remained optimal (HbA1c 38 mmol/mol) with further BW reduction (71 kg). Neither hypoglycemia nor gastro-intestinal or psychiatric adverse events were reported.

**Conclusion:**

This case supports the potential use of semaglutide for the treatment of subjects with PWS, obesity and T2D. *Ad hoc* trials are needed to evaluate the long-term efficacy and tolerability in these subjects.

## Introduction

Prader-Willi syndrome (PWS) is a complex, rare multisystem genetic disorder with an estimated prevalence of 1/10,000–1/30,000 (1). There are three main classes of chromosomal abnormalities accounting for PWS: paternal 15q11–q13 deletions (60% of cases), maternal uniparental disomy (UPD) of chromosome 15 (36% of cases) or an imprinting defect (4% of cases) (2).

A complex hypothalamic-pituitary dysfunction is currently thought to be responsible for the entire PWS phenotype with implications on the endocrine and neurologic systems (3). Individuals with PWS have an increased risk of metabolic complications, including severe obesity (4). In the Italian PWS registry, morbid obesity was observed in 54.5% of individuals enrolled in 2019 – 2020 period (5). Moreover, PWS is often associated with type 2 diabetes (T2D), that occurs in 10-25% of PWS subjects, usually in adulthood (6). The management of obesity and T2D in these subjects is still a matter of debate and no specific treatment has been established yet.

Due to their concomitant effect on glycemic control and body weight (BW), Glucagon-like Peptide 1 receptor agonists (GLP-1 RA) may represent an appealing option. Yet, literature is still limited to few reports of PWS subjects treated with exenatide or liraglutide (7–18), and with no more that 5 cases (19, 20) using with once weekly semaglutide as an add-on therapy. To the best of our knowledge, no data are currently available on semaglutide monotherapy in PWS. We herein describe the efficacy and safety of 24 months of once-weekly semaglutide monotherapy in a young Caucasian male with PWS, obesity and T2D.

## Case description

**Figure 1:**
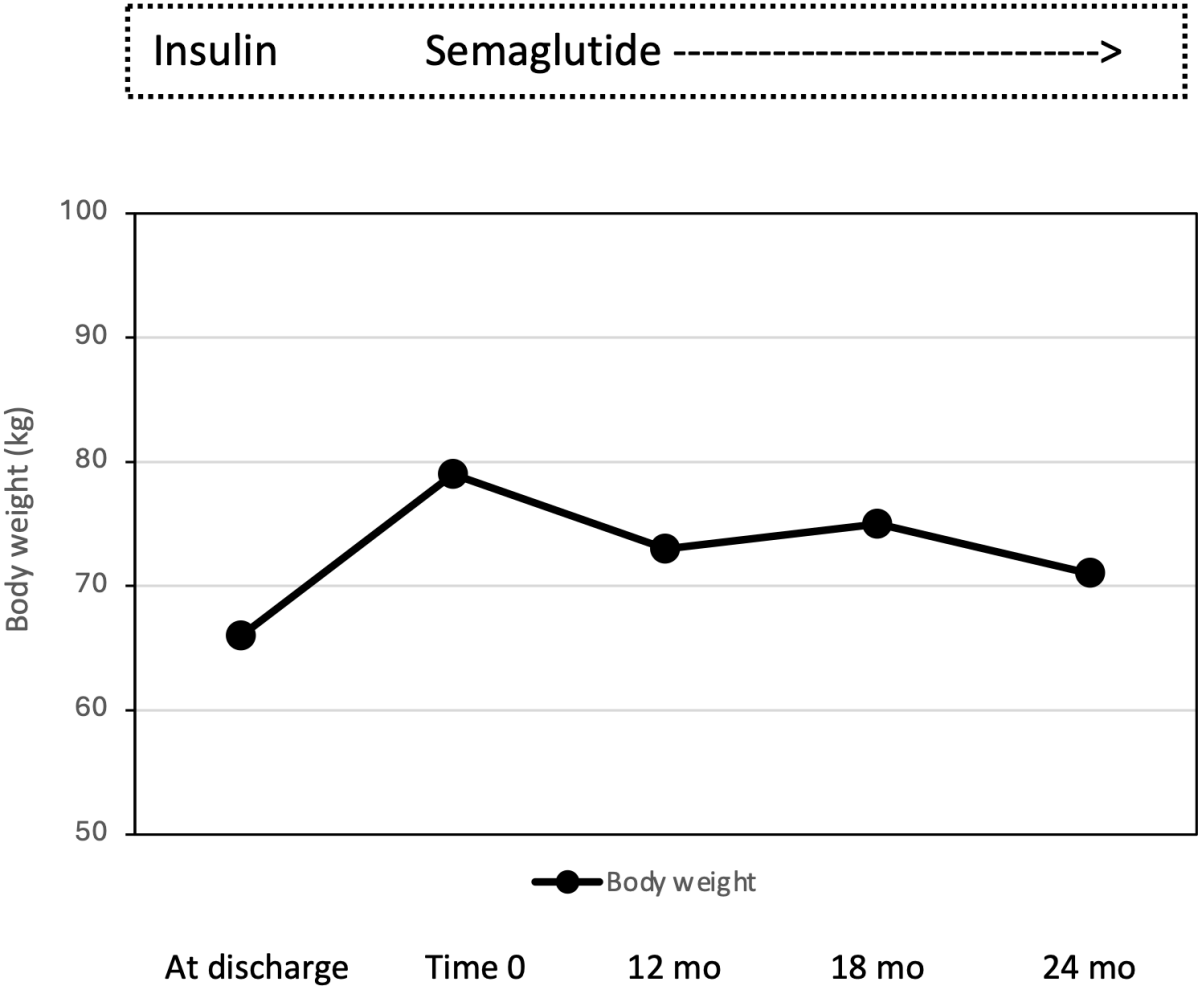
Effect of semaglutide monotherapy on body weight from time 0 to 12, 18 and 24 months (mo).

**Figure 2:**
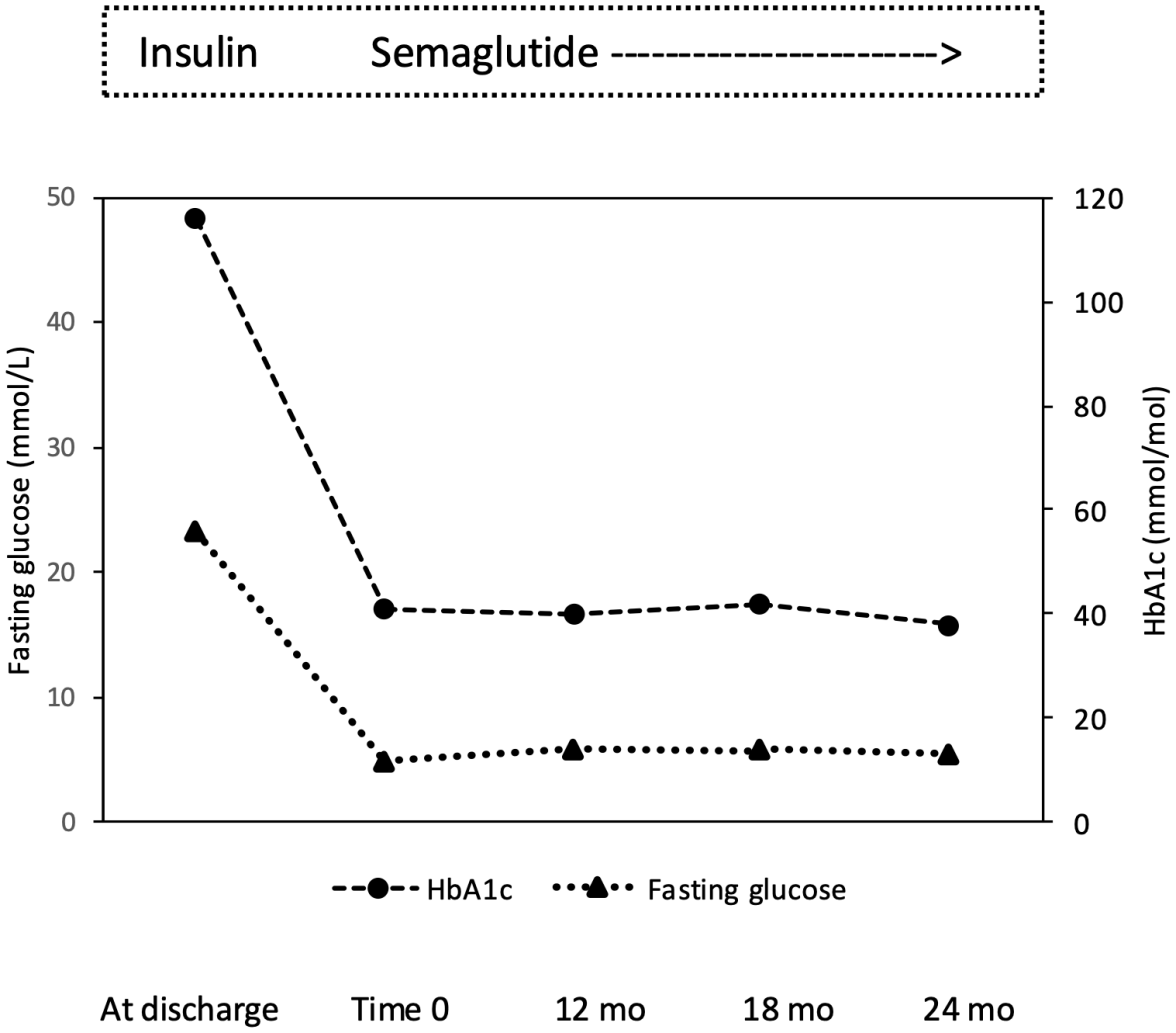
Effect of semaglutide monotherapy on fasting plasma glucose and HbA1c from time 0 to 12, 18 and 24 months (mo).

In December 2019, a 27-years-old Caucasian male was admitted to the Emergency Department of the University Hospital of Pisa for polyuria and polydipsia in the past 4-6 weeks, associated with fever and vomiting in the last 5 days. Upon admission, lab tests documented severe hyperglycemia (fasting plasma glucose 22.5 mmol/L) with no evidence of acidosis (blood gas analysis: pH of 7.42, pO_2_ 59 mmHg, pCO_2_ of 37 mmHg, HCO3- of 24 mmol/L). Serum C-reactive protein level was 9.91 mg/dl (normal value <0.5 mg/dl) and procalcitonin level 11.14 ng/mL (normal value <0.05 ng/mL). Chest X-rays revealed reticular and micronodular interstitial involvement with pleural effusion at the bases, suggestive of pneumonia. The patient was transferred to our Section of Diabetes and Metabolic Diseases with a diagnosis of *severe hyperglycemia in new-onset diabetes mellitus and sepsis*. On admission to the ward, the patient was conscious. The clinical picture was characterized by dysmorphic facies with narrow minimal frontal diameter, almond-shaped eyes and thin upper lip, small hands and feet, scoliosis, gynecomastia, testicular hypoplasia, and bilateral lower limb swelling. His BW was 66 kg, and height was 148 cm, with a body mass index (BMI) of 30.1 kg/m^2^. Systolic blood pressure was 125 mmHg, diastolic blood pressure 80 mmHg, pulse rate 90 beats per minute, body temperature 38,5°C. The rest of the physical examination was unremarkable. Fasting plasma glucose (FPG) was 23.3 mmol/L and HbA1c 116 mmol/mol. The search of islet autoantibodies (i.e., glutamic acid decarboxylase autoantibodies, anti-GAD and islet tyrosine phosphatase autoantibodies, anti-IA2) was negative. Urine albumin to creatinine ratio (ACR) in a spot urine sample was 104.5 mg/g (normal value < 30 mg/g). Liver and renal function tests were within the normal range. Insulin-like Growth Factor 1 (IGF-1) values were within the lower limits of the reference range for age (96.6 µg/L). Gonadotropin and testosterone levels were consistent with mixed hypogonadism (FSH 22.6 UI/L; LH 7.1 UI/L; testosterone 1.69 μg/L; sex hormone binding globulin, SHBG, 42 nmoL/L), while thyroid and adrenal function was normal (data not shown). Blood cultures were positive for *Candida glabrata*. Microscopic urinalysis excluded urinary tract infections. Screening for retinopathy, hypertension and cardiovascular disease was uneventful. No foot problems were observed. Abdominal ultrasound was normal, while testicular ultrasound described small testes with hypoechoic echotexture and breast ultrasound confirmed the presence of true gynecomastia. Bone mineral density (BMD), measured by dual-energy X-ray absorptiometry (DXA) at the lumbar spine, femoral neck and total hip, was consistent with osteopenia (lumbar Z-score -2.3, femoral neck Z-score -2.3, total hip Z-score -2.2). Familial history was negative for neurodevelopmental delay, genetic syndromes, and diabetes mellitus. Consanguinity was not present in the family. The patient was treated with i.v. fluid and insulin infusion, and caspofungin was started to target *C. glabrata*. Upon hyperglycemia and sepsis resolution, a basal-bolus insulin therapy was initiated (total daily insulin requirement 0.4 unit per kg of BW, approximately 26 U/day) yielding a progressive improvement of daily glucose profiles. Insulin was subsequently managed by the parents because the patient was not self-sufficient. At discharge, the patient was equipped with the FreeStyle Libre Flash Glucose Monitoring System (Abbot Diabetes Care, Alameda, California, USA). In the meantime, a PWS was suspected and, after obtaining written informed consent from both parents, the genetic test was performed. The methylation-specific multiplex ligation-dependent probe amplification (MS-MLPA) identified 2 copies of chromosome 15q11, and an abnormal DNA methylation with 2 methylated copies. Short tandem repeat (STR) linkage analysis excluded uniparental disomy and suggested the imprinting defect by epimutation. At discharge the patient was prescribed testosterone but no GH replacement therapy, in accordance with the Endocrinology consultant. Because of the COVID-19 pandemic, the patient was only seen in the outpatient clinic in March 2022 when glycemic control was at target (FPG 4.89 mmol/L; HbA1c 41 mmol/mol) despite discontinuation of the Flash Glucose Monitoring. Several non-severe hypoglycemic events were reported. A 13 kg body weight gain (from 66 to 79 kg with a BMI of 36 kg/m^2^) was observed. Given the poor adherence to diet and family burden in the management of insulin treatment and in the light of negative antibodies, insulin therapy was discontinued, and once-weekly semaglutide started at the initial dose of 0.25 mg per week, gradually increased to 0.5 mg per week. No metformin was given due to the swallowing difficulties of the patient. Twelve months after starting semaglutide, BW dropped from 79 to 73 kg (absolute change -6 kg; -7.6%; **Figure 1**), with a final BMI of 33.3 kg/m^2^, and glycemic control remained at target (FPG 5.9 mmol/L; HbA1c 40 mmol/mol; **Figure 2**). Neither hypoglycemia nor side effects were reported. At 18-month follow-up, a slight weight gain was observed (+ 2 kg; 75 kg; **Figure 1**) without deterioration in glycemic control (FPG 5.9 mmol/L; HbA1c 42 mmol/mol; **Figure 2**). Semaglutide was continued at 0.5 mg once weekly, with a personalized physical activity program and nutritional intervention. To support family members in diabetes management, FreeStyle Libre 2 sensor was also prescribed. The 14 days ambulatory glucose profile (AGP) before the 18-month visit showed a time in range (TIR) of 94%, a time below range (TBR) of 3%, and a time above range (TAR) of 3%, time with active data 71%, glucose management indicator (GMI) 41 mmol/mol and coefficient of variation (CV) 23.5%. After 24 months semaglutide treatment, BW was 71 kg (absolute change -4 kg vs. 18-month follow-up and -8 kg vs. baseline; **Figure 1**) with persistent optimal glycemic control (FPG 5.4 mmol/L; HbA1c 38 mmol/mol) (**Figure 2**). No foot, micro- and macrovascular complications were observed. Urine ACR was within the normal range (11.4 mg/g). No hypoglycemia nor gastro-intestinal or psychiatric adverse events were reported; adherence to semaglutide was optimal and caregivers’ satisfaction high.

## Materials and methods

Blood samples were obtained between 8 and 9 a.m. after an overnight fast. Urine samples were collected as first morning spot urine sample. All laboratory and hormonal parameters were determined according to standard methods. Anti-GAD and anti-IA2 autoantibodies were analyzed by a radioimmunoassay using a commercial kit (Medipan, Berlin, Germany). GFR was estimated from creatinine by the Chronic Kidney Disease Epidemiology Collaboration (CKD-EPI) equation (21). Albuminuria was evaluated by measuring the ration between albumin concentration in milligrams by creatinine concentration in grams in a spot urine sample. Multiplex ligation probe amplification was applied to identify abnormal methylation of the PWS region of chromosome 15. MLPA reagents were obtained from MRC-Holland (Amsterdam, The Netherlands; SALSA MLPA kit ME028). Data analysis was performed with Coffalyser.Net software obtained from MRC-Holland (Amsterdam, The Netherlands). The proband and his parents were genotyped for 9 short tandem repeats (STR) located in the typical PWS and Angelman syndrome (AS) deletion region and for 3 STRs located in other chromosomes by polymerase chain reaction amplification and separation on an automated ABI-3500 DNA sequencer. The polymorphic markers were analyzed by GeneScan3.1 software (Applied Biosystems, Foster City, CA, USA). The location of the STRs was obtained from UCSC Genome Bioinformatics (https://genome-euro.ucsc.edu; build 37/hg19).

## Discussion

To the best of our knowledge, this is the first case to evaluate the efficacy and safety of once-weekly semaglutide monotherapy in a young subject with PWS, obesity, and T2D.

In PWS, controlling weight and T2D remains critical to mitigate associated morbidity and mortality. However, no definitive treatment strategy has been established (22). GLP-1 RA have been shown to be quite effective in lowering plasma glucose levels with no risk of hypoglycemia and to favor body weight reduction (23). Because of the latter, GLP-1 RA, in particular liraglutide and semaglutide, are currently indicated for the pharmacologic treatment of obesity in individuals with and without diabetes (24). A such GLP-1 RA may be a therapeutic option in PWS subjects, yet the literature on this use is still scanty. The efficacy and safety profile of exenatide and liraglutide in PWS have been explored in few studies (25) and, to the best of our knowledge, the use of semaglutide as an *add-on* to other antidiabetic drugs has been reported only in 5 cases of PWS (19, 20). We now report data on the efficacy and safety of once-weekly semaglutide monotherapy in a young adult with PWS, obesity and T2D showing a meaningful body weight reduction, attainment of optimal glycemic control, good tolerability and no hypoglycemic events.

The use of exenatide and liraglutide in PWS has resulted in high variability of body weight changes, with BMI reduction ranging from 1.5 to 16.0 kg/m^2^ (25) or without significant effects, as showed in a recent trial that investigated the effect of liraglutide on weight management in children and adolescents with PWS and obesity (26). On the other hand, obesity in PWS subjects shows distinct phenotypic and metabolic characteristics that are not common to simple obesity (1). Moreover, several underlying mechanisms have been hypothesized (4), probably accounting for the variability in response to therapies.

In the case reported by Sani et al. (19), where semaglutide was added to insulin therapy, after 12 months, body weight went from 99.5 to 94.3 kg, along with a reduction in fat mass and insulin requirements. More heterogeneous are the weight loss reported by Giménez-Palop et al. (20). In our case, semaglutide (0.5 mg per week) allow body weight to go from 79 to 71 kg with an absolute change -8 kg, i.e., -10.1%. Given that a ≥5% weight loss is currently considered as clinically meaningful, our results are then of solid clinical relevance. The variability between our study as well as among the few reported observation should not surprise given the interindividual variability among different GLP-1 RA (27); rather all the so far available evidence support the potential beneficial effect of this class of agents in PWS. In our patient, along with marked body weight reduction, semaglutide monotherapy was highly effective in controlling plasma glucose levels over 2-year treatment. Moreover, we replaced insulin by once weekly semaglutide yet ensuring excellent glucose metabolic profile in a person fully dependent and with intellectual disability, without increasing the risk of hypoglycemic events and the burden to treatment management for the family members. Our subject had no sign of diabetes complication at the time of diagnosis apart from an increased ACR. Interestingly enough, ACR was normalized at the end of the observation period which may reflect the reduction in body weight and the persistent glycemic control as obtained with semaglutide, although a renal protective effect of semaglutide has been demonstrated in randomized clinical trials (28).

There are some limitations to this study. First, this is a single case report, thus, the results may be partially different from other studies and should be interpreted with caution. Second, changes in body composition were not assessed during the study. As a final limitation of the study, circulating levels of orexigenic and anorexigenic hormones were also not evaluated. However, unless of important clinical reasons, the assessment of body composition and the evaluation of hormone milieu in a single case may be limited by a variety of reasons (i.e., radioprotection, economic reasons, lack of facilities).

## Conclusion

In conclusion, this PWS case report shows a persistent effect of semaglutide 0,5 mg as monotherapy on body weight reduction and glycemic control. Our case differs to some extent with what obtained in other PWS cases highlighting the need of randomized controlled trials exploring long-term efficacy and safety of semaglutide, even at higher doses, in these patients. Understanding the role of GLP-1 RA in PWS may offer a new and effective therapeutic opportunity for a condition still orphan of an evidence-based recommendation for the treatment of PWS with obesity and type 2 diabetes.

## Data Availability

The raw data supporting the conclusions of this article will be made available by the authors, without undue reservation.
